# Olive Fruit Fly Symbiont Population: Impact of Metamorphosis

**DOI:** 10.3389/fmicb.2022.868458

**Published:** 2022-04-18

**Authors:** Catarina Campos, Luis Gomes, Fernando T. Rei, Tania Nobre

**Affiliations:** ^1^Laboratory of Molecular Biology, MED – Mediterranean Institute for Agriculture, Environment and Development, Instituto de Investigação e Formação Avançada, Universidade de Évora, Évora, Portugal; ^2^MED – Mediterranean Institute for Agriculture, Environment and Development, Instituto de Investigação e Formação Avançada, Universidade de Évora, Évora, Portugal; ^3^Laboratory of Entomology, MED – Mediterranean Institute for Agriculture, Environment and Development, Instituto de Investigação e Formação Avançada, Universidade de Évora, Évora, Portugal

**Keywords:** *Bactrocera oleae*, symbionts, metabarcoding, metamorphosis, ***Ca***. Erwinia dacicola, olive fruit fly, symbiosis-based management

## Abstract

The current symbiotic view of the organisms also calls for new approaches in the way we perceive and manage our pest species. The olive fruit fly, the most important olive tree pest, is dependent on an obligate bacterial symbiont to its larvae development in the immature fruit. This symbiont, *Candidatus* (*Ca*.) Erwinia dacicola, is prevalent throughout the host life stages, and we have shown significant changes in its numbers due to olive fruit fly metamorphosis. The olive fruit fly microbiota was analyzed through 16S metabarcoding, at three development stages: last instar larvae, pupae, and adult. Besides *Ca*. E. dacicola, the olive fruit flies harbor a diverse bacterial flora of which 13 operational taxonomic units (grouped in 9 genera/species) were now determined to persist excluding at metamorphosis (*Corynebacterium* sp., *Delftia* sp., *Enhydrobacter* sp., *Kocuria* sp., *Micrococcus* sp., *Propionibacterium* sp., *Pseudomonas* sp., *Raoultella* sp., and *Staphylococcus* sp.). These findings open a new window of opportunities in symbiosis-based pest management.

## Introduction

The olive fruit fly, *Bactrocera oleae* (Rossi, 1790), has specialized to become monophagous and it remains the most important olive crops’ pest. It is responsible for both quantitative and qualitative relevant production losses. This insect is thought to have originated in Africa and then spread to the Mediterranean basin and South Central Asia (Nardi et al., 2005). The plant–insect interaction is of dynamic nature. The most common interaction involves insect herbivory and plant defenses against these insects (Gatehouse and Gatehouse, 2002), and this predator–host relationship leads to a more or less tight coevolutionary history depending on the insects’ plant host range.

Whereas the adult of the olive fruit fly feeds on various substrates such as nectar, honeydew, fruit and plant exudates, bacteria, and even bird feces (Christenson and Foote, 1960; Drew et al., 1983; Drew and Yuval, 2000; Sacchetti et al., 2014), the larvae feed exclusively on the olive fruit tissue, digging tunnels through the pulp. As an anti-herbivory defense, the olive plant synthesizes phenols that accumulate in fruit tissues during growing and ripening, mainly the phenolic secoiridoid β-glucoside known as oleuropein (Soler-Rivas et al., 2000). To cope with these olive-plant-abundant secondary metabolites, particularly the defensive compound oleuropein, the olive fruit fly evolved to harbor a vertically transmitted and obligate bacterial symbiont—*Candidatus* (*Ca*.) Erwinia dacicola (Ben-Yosef et al., 2015). This symbiont is allocated in a specific cephalic organ, called the esophageal bulb or pharyngeal bulb, and is passed maternally to the following generation at the oviposition (Petri, 1909; Capuzzo et al., 2005; Estes et al., 2009). This mode of symbiont transmission allows for a stronger alignment of interest between the partners, and while providing the offspring with the symbiont, it also provides an opportunity for host–symbiont coevolution. Theory suggests that holometabolous insects, such as the olive fruit fly, are less likely to evolve strictly vertical transmitted symbiosis as complete metamorphosis, and the divergence of life stages poses an extra barrier to symbiont vertical transmission (Hammer and Moran, 2019).

The insect’s internal environmental conditions and resources are largely controlled by the host, providing a (more) isolated environment, and thus are less directly susceptible to changes in the outside habitat. On the other hand, the physicochemical conditions influenced by different gut compartments can display extreme gradients of oxygen, hydrogen, and pH. Far from simple, the gut environment of holometabolic insects is disrupted upon molting. The internal reorganization required by complete metamorphosis can have drastic consequences for the gut microbiota (Hammer and Moran, 2019) but also enables restructuring of the microbiota and thus may represent a special case of adaptive decoupling (Johnston and Rolff, 2015; Rolff et al., 2019).

To persist between host generations, symbionts may need to transition between intra- and extracellular phases and eventually colonize new structures (Hammer and Moran, 2019). This is indeed the case of *Ca*. E. dacicola, which transits between intra- and extracellular lifestyles during specific stages of the host’s life cycle (Estes et al., 2009, 2012). However, we do not know how the *Ca*. E. dacicola population is affected by these transitions. One could hypothesize a drop in population size from larvae stage to pupa and adults, as this symbiont’s main attributed function is aiding the larvae of the olive fruit fly in utilizing the olive pulp (Ben-Yosef et al., 2015).

Besides this particular relation with *Ca*. E. dacicola, olive fruit flies share diverse bacterial relationships with other fruit flies (Tephritidae, subfamilies Dacinae, and Trypetinae). Traditional microbiological approaches have identified other bacteria of the genera *Lactobacillus*, *Micrococcus*, *Pseudomonas*, *Streptococcus*, *Citrobacter*, *Proteus*, *Providencia*, *Enterobacter*, *Hafnia*, *Klebsiella*, *Serratia*, *Pantoea*, and *Xanthomonas* (e.g., Yamvrias et al., 1970; Fitt and O’Brien, 1985; Konstantopoulou et al., 2005; Tsiropoulos, 2009; Noman et al., 2020). More recently, molecular analyses have established the presence of *Acetobacter tropicalis*, *Pseudomonas putida*, and *Asaia* sp., *Enterobacter* sp., and *Tatumella* sp. (Sacchetti et al., 2008; Kounatidis et al., 2009; Blow et al., 2020). These studies covered different geographical areas, and not all bacteria were found in all regions. Hence, these bacteria, other than the obligatory endosymbiont *Ca*. E. dacicola, are likely to be acquired from the environment during feeding and they probably inhabit the gut. These and other bacterial microbiota can be putatively important in the olive fruit fly life cycle, but their role requires confirmation. Apart from eventual transitory species with no direct role on host fitness, some might be considered facultative symbionts. How do they respond to the metamorphosis process? Are some of these bacterial symbionts prevalent throughout the host life cycle? In the same way than for *Ca*. E. dacicola, it is thus relevant to know whether these species show any specificity for a particular development stage and why. The molting of immature insects can provoke a drastic shift of the gut microbiota, as insects typically shed the lining of the foregut and hindgut and eliminate most or all gut contents with each molt. This is a challenge for the microbiota but as discussed it also offers the opportunity to reshape the bacterial symbiotic community. Knowing this dynamics can provide tools toward the development of symbiosis-based approach to manage the olive fruit fly as a pest (Nobre, 2019; Bigiotti et al., 2021).

The present work looks thus into the dynamics of *Ca*. E. dacicola numbers at the larval, pupae, and adult stage (*via* a targeted real-time PCR approach) and at the bacterial microbiota present at these three development stages of the olive fruit fly (*via* 16S metabarcoding). It attempts to define a “core microbiome” of bacterial symbionts, other than *Ca*. E. dacicola, that prevail to the drastic changes imposed by metamorphosis. By this, it intends to open new perspectives on other potential symbionts to be considered for applied management of the host pest.

## Methodology

### Sampling

Olive fruits with signs of fruit fly infestation were collected at a single location from two local cultivars—“Galega” (considered highly susceptible to olive fruit fly) and “Redondil.” The trees were within a commercial olive orchard at the Herdade Álamo de Cima (38°29′49.44′′ N, 7°45′8.83′′ W) in southern Alentejo, Portugal. Per cultivar, the collected olives were randomly allocated in three different plastic boxes for collection of larvae, pupae, and adults. While pupae and adults were gathered from naturally emerging larvae, the collection of larvae implied the dissection of the fruit to remove the larvae (3rd instar) feeding on the fruit pulp. Up to 10 individuals per cultivar per development stage were collected and stored at −20°C in 70% ethanol until DNA extraction. Individuals were allowed to dry on filter paper prior to DNA extraction. To provide for surface sterilization, this was done under UV light in a closed chamber for 5 min. DNA from the whole body tissue was extracted using the ZymoBIOMICS DNA Kit (Zymo Research^®^, Irvine, CA, United States), claimed to be more suitable for an unbiased DNA extraction for microbiome profiling.

### *Candidatus* Erwinia dacicola Identification and Quantification by Real-Time PCR

The absolute quantification of *Ca*. E. dacicola was performed by qPCR using SYBR Green chemistry, on 10 individuals per cultivar and per developmental stage. The forward primer used was the specific (EdF1) previously described (Estes et al., 2009) (5′-CTAATACCGCATAACGTCTTCG-3′) and the reverse primer was designed based on the *Ca*. E. dacicola 16S sequences available from the same geographical area (Nobre, 2021) and on a selection of sequences deposited on GenBank (5′-TCATCCTCTCAGACCAGCTA-3′).

The qPCR plates were run in a LineGene 9600 Plus System (BIOER, Hangzhou, China) using two technical replicates per sample. An 18-μl reaction mixture was used, using 9 μl of SYBR Green [NZYSupreme qPCR Green Master Mix (2×) ROX, Nzytech, Portugal], 0.5 μl of the reverse and forward primers (10 μM), 7 μl of H_2_O, and 1 μl of DNA. The quantification cycle (Cq) values were acquired for each sample with the following cycling conditions: 10 min at 95°C for initial denaturation, an amplification program of 40 cycles at 95°C for 15 s, and 60°C for 1 min. The fluorescence threshold was manually set above the background level. No template controls were included in all plates. The specificity of qPCR reactions was evaluated by melting curve analysis.

Specific amplification of target DNA was confirmed by cloning PCR amplicons into pGem R-T Easy vector (Promega, Madison, WI, United States) and used to transform *Escherichia coli* JM109 (Promega) competent cells, by standard methodologies, and sequenced through Sanger procedure. Clones were identified by Blast against the NCBI database.

To determine the amplification efficiency of the primers, a standard curve was generated from a sevenfold dilution series of plasmid DNA, used to draw a calibration curve in the dynamic range chosen (8E1 to 8E7 target copies). Amplification efficiencies were calculated through the equation *E* = (10(–1/slope) – 1) × 100, as well as slope and linearity (coefficient of determination, R2). The method performed for absolute DNA quantification was based on the determination of the absolute number of target copies previously described by Campos et al. (2018).

### Generation of 16S Amplicons and Data Processing

Extractions above were pooled per state of development (larval, pupal, and adult) and cultivar (“Galega” and “Redondil”) in such a way that each sample for metabarcoding consisted of a mixture of DNAs extracted from 10 olive fruit flies. The next generation sequencing (NGS) sequencing procedures were performed at STABVIDA, Lda (Portugal). After quality control of the DNA, to ensure samples had sufficient integrity and quantity for optimal amplification, the library construction was performed using the Illumina 16S Metagenomic Sequencing Library preparation protocol and the generated DNA fragments (DNA libraries) were sequenced with MiSeq Reagent Kit (Illumina, San Diego, CA, United States) v3 in the lllumina MiSeq platform, for the V3 and V4 regions of the 16S rRNA gene, using 300 bp paired-end sequencing reads (available at NCBI, BioProject PRJNA800389).

Bacterial amplicons were processed and analyzed using the Galaxy mothur Toolset.^1^ The recommended workflow was followed, and bacterial operational taxonomic units (OTUs) were picked at 97% similarity. Taxonomies were determined using the SILVA reference database.^2^

## Results

**FIGURE 1:**
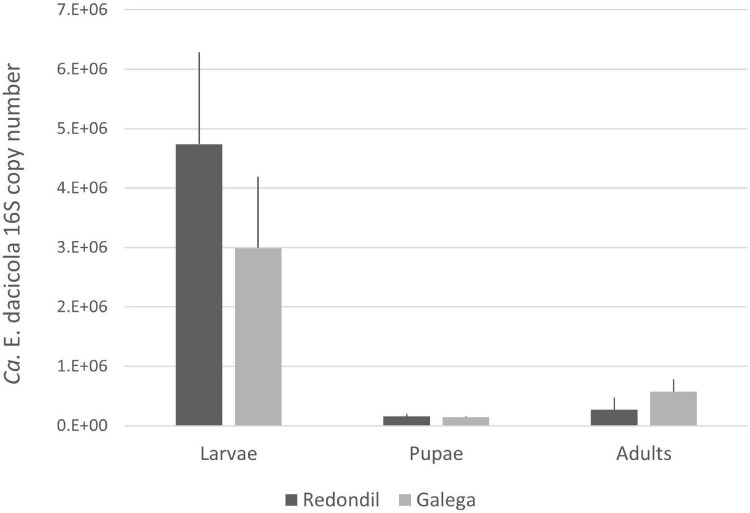
Average number of 16S *Candidatus* Erwinia dacicola copies (and standard error) estimated 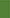 Redondil 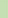 Galega.

The average number of 16S *Ca*. E. dacicola copies between flies sampled from both cultivars were not significantly different in each development stage (Figure 1; probability associated with a Mann–Whitney *U*-test: 0.667 for larvae, 0.668 for pupae, and 0.103 for adults). A Kruskal–Wallis test was performed to determine if median values of 16S *Ca*. E. dacicola copies were the same for the three different development stages of the host, as retrieved feeding on each of the olive cultivars. The test showed that the median number of 16S copies of this symbiont was not the same (Redondil, *H* = 12.740, *p* = 0.002; Galega, *H* = 11.445, *p* = 0.003) among larvae, pupae, and adults.

**FIGURE 2:**
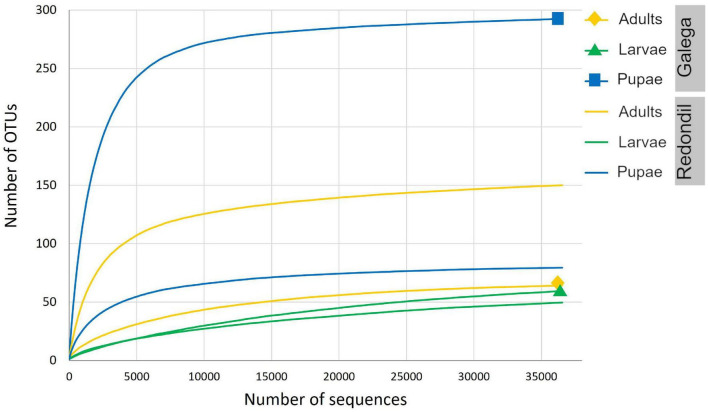
Rarefaction curves showing the number of bacterial OTUs for each sample, defined at the 97% sequence similarity cutoff in mothur, relative to the number of total sequences.

Sequencing of the samples generated from 192 974 to 216 126 raw sequence reads, which is in accordance with the expected output. After denoising, a total of 372 unique features (OTUs) of 222 bp were be detected, even though the total amount of OTUs observed per sample varied considerably (also initial DNA concentration and integrity was different between samples, which might have led to this output differences), and the alpha-rarefaction curve clearly reached a plateau, indicating that the sequencing was deep enough to detect present 16S diversity (Figure 2). Almost all reads (99.83%) were associated with bacteria.

**FIGURE 3:**
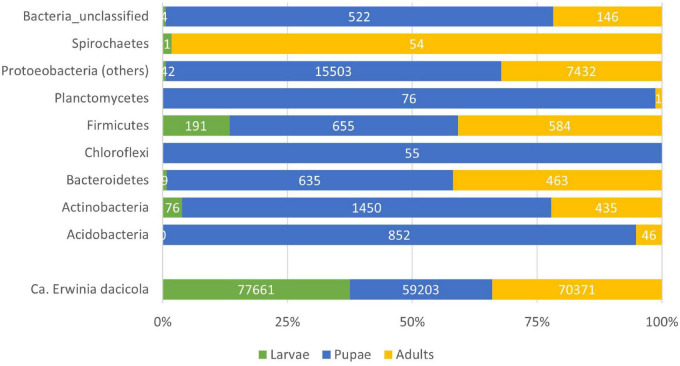
Barplot of the total number of reads considering referred to a given Phyla within the bacteria. The Proteobacteria *Candidatus* Erwinia dacicola is represented separated from the other members of the Phylum due to its predominance in the bacterial microbiota.

For the rest of the analysis and having in mind the possible significance of OTUs presence, we have considered a more rigid threshold of 25 reads per OTU across development stages and cultivar; the number of valid reads remained comparable between larvae (78,084 reads), pupae (78,951 reads), and adult (76,162 reads) stages. As expected, the presence of the obligate symbiont *Ca*. E. dacicola dominates the reads in all the development stages (Figure 3). In the larval stage, however, it comprises 99% of the 16S sequences, followed by the adult stage with 92% of the bacterial reads being of *Ca*. E. dacicola.

In total, 18 OTUs (comprising 672 reads) were not possible to classify within the bacteria domain. Within the Acidobacteria, 25 OTUs of uncertain placement were recorded, within four groups: Gp1 *incertae familiae* (16 OTUs, 556 reads), Gp2 *incertae familiae* (2 OTUs, 91 reads), Gp3 *incertae familiae* (6 OTUs, 220 reads), and Gp7 *incertae familiae* (1 OTUs, 31 reads). However, the pool of pupae from Galega variant (MP) is responsible for almost 95% of these reads. Twenty OTUs were recorded for the Actinobacteria, comprising 1,961 reads. Only 1 OTU Coriobacteriales was recorded, an unclassified Coriobacteriaceae mainly from the adults’ sample from Redondil (RA-28 reads of 33 reads). A *Conexibacter* sp. (Solirubrobacterales) was recorded, but only in the MP. The Actinomycetales (18 OTUs, 1,896 reads) was the most recorded order within the Actinobacteria. Three of the 18 OTUs remained unclassified within the Actinomycetales. The MP sample was responsible for several OTUs, namely, a *Brachybacterium* sp., a *Friedmanniella* sp., and a *Kineococcus* sp. *Dermacoccus* sp. is recorded in the pupae (62 records, 39 records in MP plus 23 in RP, against 8 in MA and 2 in RL).

**FIGURE 4:**
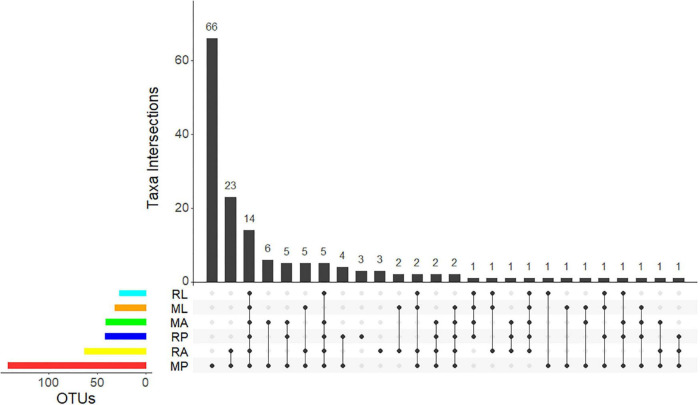
UpSet plot summarizing the presence of OTUs in the different olive fly samples. The bottom left horizontal bars shows the total number of OTUs per sample. The circles in the panel’s matrix represent the unique and common parts in Venn diagram sections (unique and overlapping OTUs). Connected black circles indicate a certain intersection of OTUs between treatments, while grey circles show no intersection. The top vertical columns summarize the number of OTUs for each unique or overlapping combination. M stands for “Galega” cultivar and R for “Redondil” cultivar, followed by A for adults, P for pupae, and L for larvae.

From the 14 bacteria shared at all development stages (Figure 4), albeit in different proportions, 5 are within the Actinomycetales: *Kocuria* sp. (7 records in the larvae, 319 in the pupae, and 33 in the adults), *Micrococcus* sp. (2 records in the larvae, 41 in the pupae, and 21 in the adults), *Propionibacterium* sp. (56 records in the larvae, 376 in the pupae, and 252 in the adults), and *Corynebacterium* spp. (2 OTUs; 6 records in larvae, 96 in the pupae, and 49 in the adults). No representative of the phylum “Bacteroidetes” (1,107 records) was present in all samples. Approximately 10% of these records (111) were not possible to further classify, and the vast majority was associated with the sample MP (76 records) and the other corresponded to 6 OTUs within the Bacteroidales (226 records of Bacteroidaceae and 1 OTU with 44 representatives of a Rikenellaceae), 3 Flavobacterium OTUs (271, 2 *Chryseobacterium* spp. OTUs and 1 *Uzinura* sp. scattered with no pattern through the samples), and 10 Sphingobacteria OTUs (all 455 records but 2 are in the MP). Also, only the MP pupae sample showed 2 OTUs of Ktedonobacteria and 2 OTUs of Planctomycetales. The only Spirochaetales found, a *Treponema* sp., was recorded in the adult sample RA. Of the 20 Firmicutes OTUs, 9 are Bacilli, 8 Clostridia, 1 Negativicutes, and 2 Tissierellia. From the 9 Bacilli, only one is shared by all samples (*Staphylococcus* sp., with 437 reads in total). A *Planomicrobium* sp. is present only in the pupae, albeit in variable low numbers (25 reads in MP and 2 in RP). Still within the Bacillales, *Neobacillus* sp. is one of the two OTUs (Figure 4) registered in the pupae (32 reads in MP and 13 in RP) and with a marginal presence in the adults (1 record in MA and 1 in RA). The remaining two Bacillales are only present on the deviating sample MP. Four Lactobacillales OTUs are registered, with no apparent presence/absence pattern. Within the Clostridia (438 reads), the 2 Eubacteriales OTUs are represented by 1 *Romboutsia* sp. in the pupae (25 reads in MP and 6 reads in RP) and 1 *Syntrophococcus* sp. (25 reads in RA), and the 6 Clostridiales OTUs are represented by 2 unclassified OTUs (together with 106 reads) and 1 Lachnospiraceae (61 reads) and 3 unclassified Ruminococcaceae (215 reads) spread between RA, ML, and MP.

**FIGURE 5:**
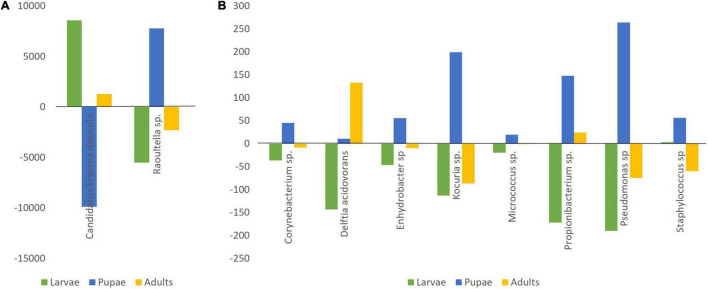
The number of reads deviating from the average number per life stage of the 14 shared OTUs, grouped in 10 genera/species. **(A)** *Candidatus* Erwinia dacicola (average 69,078 sequence reads) and *Raoultella* sp. (average 5,506 sequence reads); **(B)** *Corynebacterium* sp. (average 41 sequence reads), *Delftia acidovorans* (average 241 sequence reads), *Enhydrobacter* sp. (average 51 sequence reads), *Kocuria* sp. (average 120 sequence reads), *Micrococcus* sp. (average 21 sequence reads), *Propionibacterium* sp. (average 228 sequence reads), *Pseudomonas* sp. (average 210 sequence reads), and *Staphylococcus* sp. (average 146 sequence reads).

Additional to the 5 Actinomycetales OTUs shared by all samples, the remaining 8 of the 14 OTUs presented in all samples (Figure 5) belong to the Proteobacteria and correspond, within the Gammaproteobacteria, to 3 *Ca*. E. dacicola unique sequences (207,235 reads), followed by 1 *Raoultella* sp. (16,519 reads), 2 *Pseudomonas* sp. (629 reads), 1 *Enhydrobacter* sp. (154 reads), and within the Betaproteobacteria to 1 *Delftia acidovorans* (724 reads). Only the *Raoultella* sp. emerges with relatively high numbers of reads (though 10 × less than *Ca*. E. dacicola) but showing an opposite pattern of variance across life stages (Figure 5A).

Bacteria exclusively present on the pupae life stage (Figure 4) are the already referred to Actinobacteria *Rothia* sp. and the two Firmicutes *Romboutsia* sp. and *Planomicrobium* sp. plus Alphaproteobacteria *Sphingomonas* sp. (46 reads). No bacteria were found exclusively in the adults pooled samples nor in the larvae.

**FIGURE 6:**
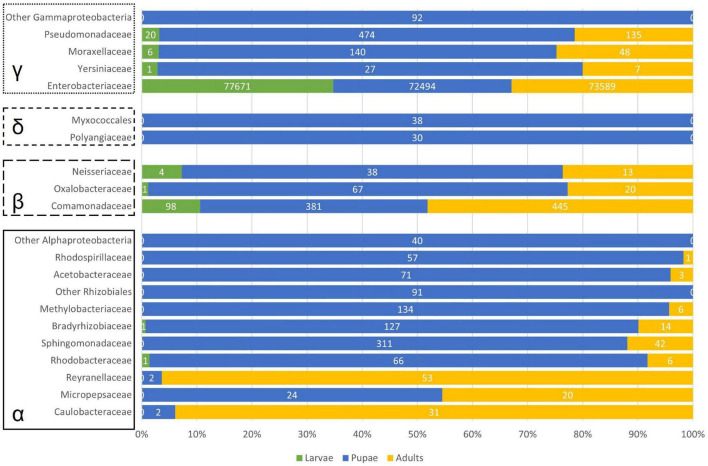
Barplot of number of reads considering the represented Proteobacteria. α – 493 Alphaproteobacteria; β – Betaproteobacteria; δ – Deltaproteobacteria; γ – Gammaproteobacteria.

In the pupal stage, other Proteobacteria than the obligate symbiont were also predominant in the samples, comprising almost 20% of the registered reads in this development stage (Figure 3). As such, Figure 6 gives an overview of the main represented groups of Proteobacteria across the olive fruit fly development stage.

Within the Alphaproteobacteria, we found 21 OTUs, of which only one could not be classified further. The families Caulobacteraceae (*Caulobacter mirabilis*, almost exclusively in the adults, Figure 6), Reyranellaceae (*Reyranella* sp.), and Micropepsaceae (*Rhizomicrobium* sp.) have only one representative. Within the order Rhizobiales, 2 OTUs remained unclassified further, while 2 OTUs of the family Bradyrhizobiaceae were recorded and 3 *Methylobacterium* sp. sequences belonging to the Methylobacteriaceae family (Figure 6). Two Rhodobacteraceae (*Paracoccus* sp. and *Tabrizicola* sp.) were recorded in low numbers and mainly associated with the sample MP. The same can be said for the 3 OTUs within the order Rhodospirillales (2 Rhodospirillaceae and 1 Acetobacteraceae). The Sphingomonadaceae are represented by 3 *Sphingomonas* sp. sequences totalizing 296 reads, of which 96% are recorded in the pupae (only 13 reads were found in the sample MA) and by 2 *Novosphingobium* sp. sequences (28 reads in MP and 29 in RA).

Betaproteobacteria were mainly represented by the above-referred *Delftia acidovorans* but 5 other OTUs were recorded within the family Comamonadaceae (*Melaminivora* sp. and almost exclusively in MP, *Ramlibacter* sp., *Alicycliphilus* sp., *Comamonas* sp., and *Delftia* sp.). Scattered through the samples, 2 *Massilia* (Oxalobacteraceae) and 2 *Neisseria* (Neisseriaceae) OTUs were registered (Figure 6). We found only two representatives of Deltabacteria, a non-identifiable Myxococcales, and a member of the Polyangiaceae family.

Apart from the obvious dominant Gammaproteobacteria *Ca*. E. dacicola, and the already noted *Raoultella* sp., *Enhydrobacter* sp., and *Pseudomonas* sp., also present in the bacterial microbiome, were *Acinetobacter* sp. (absent from ML) and *Serratia* sp. (absent from RL and RA), albeit in lower numbers.

## Discussion

*Candidatus* Erwinia dacicola is prevalent throughout the olive fruit fly life stages (Estes et al., 2012) and it resides in the larvae intracellularly within midgut cells, and in the adult within the esophageal bulb and female ovipositor diverticula but also extracellularly in the foregut (Estes et al., 2009). We have now shown that much higher population levels are kept during the larval stage, followed by a drastic drop at the pupae and then the specific symbiont population is recovered, but to levels well below of the observed when in the larval midgut cells. This seems to corroborate the crucial role of the *Ca*. E. dacicola in larval survival (Ben-Yosef et al., 2015), allowing the larvae to overcome the negative effect of oleuropein and develop feeding on the pulp of unripe olives. Nonetheless, the abundance difference found between larvae and adults is surprising as it contradicts the previously reported highest abundance of this obligate symbiont in the adults when compared to larvae (Estes et al., 2012). A word of caution should be put forward, because although effort had been put to surface sterilize the insects prior do DNA extraction, we cannot rule out that no larvae or pupae OTUs come from olive pulp. According to Estes et al. (2009), the rot tunnels that the larvae made while feeding on the pulp also contained *Ca*. E. dacicola and this might account for a part of the higher amount of this bacteria found on the larvae. However, we do not think that a putative bias due to insufficient sterilization is enough to explain the higher abundance of the obligate symbiont in the larvae when compared to the recently emerged adults. Considering insufficient surface sterilization as the solely cause raises even more questions as no free-living representatives of *Ca*. E. dacicola are known. At this stage, apart from the fact that Estes et al. (2012) and our work deal with different populations exploiting different olive cultivars, we are not able to offer any explanation for the divergence in the results. *Ca*. E. dacicola is considered needed for the survival of the larvae as, through a mechanism not yet known, this obligate symbiont promotes the detoxification of oleuropein, the main phenolic compound on the immature olive fruit.

In the adult stage, the olive fruit fly feeds on a variety of sources (Drew and Yuval, 2000), none of which is known to be particularly rich in phenolic compounds. Because maintenance costs of an effective population size of the bacteria are prone to exist, one could hypothesize that a cost–benefit balance would select for a decrease in the endosymbiotic population size of *Ca*. E. dacicola at adult stage. Indeed, our results clearly show such a population decrease in the quantification experiment and to a certain extent in the 16S metabarcoding, considering the number of reads as surrogates of the abundance. The vertical transmission mode of this obligate symbiont, *via* the female upon oviposition, implies the need of at least a residual population of the obligate symbiont at the adult stage. Therefore, *Ca*. E. dacicola needs to survive the breakdown and rebuilding of the host tissues resulting from the metamorphosis. The presence and maintenance of bacteria in the gut during metamorphosis in Diptera is already established (e.g., Bakula, 1969; Wong et al., 2011) and more evidence is being gathered for an important role of the host immune system in shaping the gut microbiota in a way that benefits the resulting adult host (Russell and Dunn, 1996; Johnston and Rolff, 2015; Malacrinò et al., 2018).

In this context, it remains to access whether the symbiotic population of *Ca*. E. dacicola would increase once the adult flies would be developing in the wild and whether that would be diet dependent. The adults of olive fruit flies seem to rely on food sources that are poor and unbalanced in their amino acid composition, in spite of its variety, and the bacteria were able to compensate for it (Ben-Yosef et al., 2010). *Ca*. E. dacicola is thus also important for adult flies, metabolizing complex nitrogen compounds and promoting fly successful reproduction (Ben-Yosef et al., 2014). Therefore, their prevalence to adult stage and maintenance of higher numbers might have a more positive benefit than initially hypothesized.

Fruit flies are known to associate with extracellular, environmental bacteria such as *Klebsiella*, *Pantoea*, and *Enterobacter* and their prevalence associated with their ability to fix nitrogen (diazotrophs) (Behar et al., 2005, 2008; Ben-Yosef et al., 2014). Fruit flies feed on extremely high C:N ratio diets and therefore the presence of diazotrophs (mostly members of the Enterobacteriaceae family) is expected (Bar-Shmuel et al., 2020). *Enterobacter* sp. was previously identified as present in all life stages of wild olive flies (Estes et al., 2009), but not in the present study. However, we found another Enterobacteriaceae - Raoultella sp.- also before referred to as associated with olive fruit fly (adult stage, Estes et al., 2009). The presence of *Raoultella* sp. needs to be highlighted due to its consistent higher numbers and presence pattern, with higher numbers in the pupal stage. This bacterium is also a diazotroph suggesting a contribution to nitrogen requirements.

Also prevailing across life stages, and apart from the obligate symbiont and the *Raoultella* sp., we could identify 12 more OTUs, grouped in 8 genera/species (recalling *Corynebacterium* sp., *Delftia* sp., *Enhydrobacter* sp., *Kocuria* sp., *Micrococcus* sp., *Propionibacterium* sp., *Pseudomonas* sp., and *Staphylococcus* sp.). From these, *Delftia* sp. is the only showing an increase in sequence number at the adult stage. This bacterium was previously sampled from insects, including several *Bactrocera* species (Prabhakar et al., 2013; Wang et al., 2014; Luo et al., 2018). In our data set, it is the only core bacteria to increase in number at the fly adult stage. It should be kept in mind that the metamorphosis occurred in laboratory conditions, with no contact with the exterior. Therefore, the registered OTUs and respective reads should be seen in the confined environment of what was already present and not what could be the adult microbiota once in the wild. In this context, it is likely that the major shifts observed are due to a combination of direct influence of the metamorphosis process and consequent bacterial population competition. In the wild, the olive fruit fly would be in contact with a diversity of bacteria of which some would become potential horizontally acquired symbionts or transient gut microorganisms.

Metamorphosis entangles drastic changes. Adding to symbiont transmission challenges, prevalence, or loss of microbially mediated phenotypes and enforced symbionts’ bottlenecks, the process of metamorphosis entails challenges which microbial symbionts can help hosts to meet (Hammer and Moran, 2019). As such, the higher levels of some bacterial symbionts in pupation stage (Figures 3, 5, 6) could be thought helping to meet nutritional demands, such as to provide amino acids and to recycle nitrogenous waste (as seen for the carpenter ant; Zientz et al., 2006; Stoll et al., 2010). Microbiota can aid host not only by providing nutrients needed to rebuild the adult body but can also protect hosts from pathogens or serve as cues triggering the onset of metamorphosis (Hammer and Moran, 2019). At the pupal stage, and as shown by Greenberg (1969) during host fly pupation, bacteria that resist mechanical and immunological exclusion will then compete intensely for colonization of the pupal gut. To better understand the bottleneck at the pupal stage, an experiment analyzing specific insect organs instead of the whole insect body should be devised, which, although not straightforward, should be possible with current laser microdissection techniques (Jones et al., 2004). The metamorphosis process and all the constraints that it encompasses imply a flexibility in the structure, abundance, and activity of insect-associated bacteria across host life stages.

The maintenance of symbionts and the gut microbiota of holometabolic species is a challenge, but metamorphosis also offers the benefit of allowing an extensive change in microbiota between the larval and adult stages (Hammer and Moran, 2019; Rolff et al., 2019). The demands and challenges faced by the olive fruit fly during the fruit pulp-confined larval stage are clearly different from the ones faced by the free-living adult feeding on available substrates such as nectar and honeydew, and they shape the bacterial microbiota mainly through exclusion at metamorphosis, competition, and likely horizontal acquisition of new symbionts. The understanding of these dynamics, and of the fundamental functions provided by the core microbiota, is a necessary step if the development of symbiosis-based pest management strategies is envisaged.

## Data Availability Statement

The datasets presented in this study can be found in online repositories. The names of the repository/repositories and accession number(s) can be found below: https://www.ncbi.nlm.nih.gov/, BioProject ID PRJNA800389.

## Author Contributions

TN conceived and supervised the study. CC and TN designed and performed the experiments, analyzed the data, and wrote the manuscript. LG, FR, and TN performed the sampling. All authors have read and approved the manuscript.

## Conflict of Interest

The authors declare that the research was conducted in the absence of any commercial or financial relationships that could be construed as a potential conflict of interest.

## Publisher’s Note

All claims expressed in this article are solely those of the authors and do not necessarily represent those of their affiliated organizations, or those of the publisher, the editors and the reviewers. Any product that may be evaluated in this article, or claim that may be made by its manufacturer, is not guaranteed or endorsed by the publisher.

## Footnotes

1
https://academic.oup.com/gigascience/article/8/2/giy166/5266305

2
https://academic.oup.com/nar/article/41/D1/D590/1069277
